# Developing a computer delivered, theory based intervention for guideline implementation in general practice

**DOI:** 10.1186/1471-2296-11-90

**Published:** 2010-11-18

**Authors:** Lisa McDermott, Lucy Yardley, Paul Little, Mark Ashworth, Martin Gulliford

**Affiliations:** 1 School of Psychology, University of Southampton, Shakleton Building, Highfield, Southampton, UK; 2 School of Medicine, University of Southampton, Aldermoor Health Centre, Aldermoor Close, Southampton, UK; 3 Department of Primary Care and Public Health Sciences, Kings College London, Capitol House, Weston Street, London, UK

## Abstract

**Background:**

Non-adherence to clinical guidelines has been identified as a consistent finding in general practice. The purpose of this study was to develop theory-informed, computer-delivered interventions to promote the implementation of guidelines in general practice. Specifically, our aim was to develop computer-delivered prompts to promote guideline adherence for antibiotic prescribing in respiratory tract infections (RTIs), and adherence to recommendations for secondary stroke prevention.

**Methods:**

A qualitative design was used involving 33 face-to-face interviews with general practitioners (GPs). The prompts used in the interventions were initially developed using aspects of social cognitive theory, drawing on nationally recommended standards for clinical content. The prompts were then presented to GPs during interviews, and iteratively modified and refined based on interview feedback. Inductive thematic analysis was employed to identify responses to the prompts and factors involved in the decision to use them.

**Results:**

GPs reported being more likely to use the prompts if they were perceived as offering support and choice, but less likely to use them if they were perceived as being a method of enforcement. Attitudes towards using the prompts were also related to anticipated patient outcomes, individual prescriber differences, accessibility and presentation of prompts and acceptability of guidelines. Comments on the prompts were largely positive after modifying them based on participant feedback.

**Conclusions:**

Acceptability and satisfaction with computer-delivered prompts to follow guidelines may be increased by working with practitioners to ensure that the prompts will be perceived as valuable tools that can support GPs' practice.

## Background

Clinical guidelines are constantly changing as decisions about best clinical practice change in line with evolving medical science [1]. However, practitioner non-adherence to guidelines has been widely identified across health services [2]. Consequently, strategies have been developed to improve adherence to guidelines in a range of clinical settings [3]. A wide variety of techniques have been used, including educational programmes and materials, patient materials, reminders and computer delivered systems [4,5].

Prompts which act as reminders of recommended standards of clinical practice have been found to improve the delivery of preventive health care services [6]. Specifically, strategies such as reminders which occur during a consultation or at the point of decision making are more likely to be effective [7,8]. Evidence has demonstrated that embedding such reminders into the flow of care and providing easy access to information can improve patient care and change healthcare professionals' behaviour [9,10].

Increasingly, the implementation of guideline reminder interventions is through the use of computer (i.e. clinical information) systems [11]. Computer-based decision support reminders for the implementation of guidelines have been found to improve clinical performance across a number of studies [12] and have been generally effective in changing health care professionals' behaviour [13,14]. In particular, computer interventions which use reminders and automatic prompts have been found to be most successful [15]. The use of such computer based interventions has led to significantly increased adherence to guidelines and improvements in health-related outcomes across a variety of conditions and behaviours including RTI, constipation, croup, urticaria, urinary tract infections, and postoperative nausea and vomiting [16-18]. The use of these interventions has been demonstrated as effective for both prescribing behaviour [16] and preventive medical care [19].

However, success rates of computer-delivered interventions have varied considerably, with prescribing improvements reported from 3% [17] to 42% [16]. Variations in success rates are likely to be due to the wide range of content involved in such computer delivered messages, which have varied from decision support [18] to simple presentation of the clinical evidence [11]. Based on inconsistent findings in the success rate of such techniques, any intervention development must focus on the specific behaviour change methods and theory which may be relevant in order to achieve optimal results [20]. A recent review concluded that computer based decision support systems can improve practitioner performance, but the effects remain understudied and inconsistent [15]. The implementation of evidence-based guidelines may sometimes be unsuccessful due to a lack of consideration of the theoretical behaviour change processes which may be involved [21]. The explicit use of behaviour change theory may therefore provide an important tool on which to base the development of interventions[22]. Application of theory may benefit the intervention development process by providing a consistent and generalisable framework and promoting understanding of components which may facilitate change for a specific behaviour [21].

Research has identified theoretical components relating directly to the effective implementation of clinical guidelines in healthcare settings [20]. Social cognitive theory proposes that the environment plays a key role in influencing an individual's behaviour [23], and that one of the most important mechanisms involved in successful behaviour change is an individual's belief in their ability to exercise control over their environment [24]. The more controllable an individual perceives their environment to be, the more likely they are to succeed in performing the desired behaviour [25], although the environment must also offer opportunities for support [26]. The importance of environment has also been supported in guideline implementation research, which has demonstrated that interventions which are embedded in a relevant environment and occur during the flow of care are more likely to succeed [7,8]. Social cognitive theory [23] proposes that self-efficacy beliefs function as key determinants of motivation for a specific behaviour. Self-efficacy refers to an individual's belief in their ability to conduct a specific behaviour. Individuals with high self-efficacy for a task are more likely to perform the behaviour. GPs' self-efficacy has been implicated as a predictor of intended adherence to recommendations for prescribing [5,20]. Social cognitive theory also suggests that anticipated outcomes ('outcome expectancies') of a behaviour influence the likelihood that it will be performed, and outcome expectancies are significantly associated with intended prescribing behaviour [5,20]. Outcome expectancies that may be relevant to prescribing decisions include anticipated patient pressure [27], beliefs about risks and benefits associated with characteristics of the disease and credibility of the guideline source and content [28].

Therefore, an intervention which creates a controllable and supportive environment, increases self-efficacy, promotes positive outcome expectancies and reduces negative outcome expectancies might support better GP adherence to guidelines. In addition, the inclusion of these factors in a computer delivered reminder intervention may be an optimal mode of delivery. Furthermore, the use of qualitative research can provide an integral component in the development of an intervention, which can help to clarify the mechanisms through which the intervention works, identify potential barriers to change [29], provide information on the individual needs of users [30], and explore relevant issues which can be used to further develop and refine the intervention model [31].

A lack of adherence and need for guideline implementation has been reported in relation to both antibiotic prescribing for RTI and recommendations for the prevention of secondary stroke [4,32,33]. These provide two contrasting conditions for which to target an intervention, as RTIs are usually brief and self-limiting [34] in comparison to stroke, in which patients experience a less frequent, potentially life threatening long-term condition (involving both functional impairment and increased risk of subsequent cardiovascular events) [35].

### Aims

The aim of the present study was to develop theory informed, computer-delivered interventions intended to promote adherence to guidelines by presenting GPs with prompts during the consultation. The aim was to produce prompts which GPs would view as feasible and acceptable in practice. The intervention was developed to be assessed in a trial which follows.

The prompts were designed to a) promote adherence to antibiotic prescribing recommendations in accordance with the NICE guidelines [32] (promote no antibiotic prescribing, or delayed antibiotic prescribing, instead of the immediate prescription of antibiotics for RTI) and b) promote adherence to recommendations from the Intercollegiate Stroke Working Party for secondary prevention of stroke [36].

The development of prompts was informed by both theory and feedback from qualitative interviews with GPs. The aim of the interviews was to identify factors and characteristics likely to influence adherence to the guideline behaviours, in order to inform development and refinement of prompts.

## Methods

### Design of study

The study used a qualitative design involving both semi-structured and 'think-aloud' interviews with 33 GPs. Face-to face interviews lasting approximately 40 minutes were conducted in GP surgeries. In the first stage 22 semi-structured interviews were conducted using paper-based prompts. The second stage involved 11 'think aloud' interviews using computer-based prompts. All interviews were recorded using a digital voice recorder, and were fully transcribed.

### Participants

Participants were 33 GPs from practices across the south of England (these included both inner-city and rural locations). The primary care trusts which were recruited from include Southampton City, Hampshire, Portsmouth, Bournemouth and Poole, Wiltshire, Lambeth, Southwark, and Lewisham. The surgery size varied widely across practices with the number of full time or equivalent GPs ranging from 1 to 11, and the number of patients registered to each full time equivalent GP ranging from 826 to 2896. The index of multiple deprivation score (IMD) also varied greatly, and ranged from 2 to 43. The primary care research network (PCRN) assisted in recruitment and contacted participating practices via fax/news letter. Consecutive GPs responding to the study invitations were recruited to take part. Written informed consent was obtained prior to each interview.

### Procedure

The study was approved by the London - Surrey Borders REC and received PCT R&D approval (09/H0806/7). A semi-structured interview was designed to identify factors likely to influence successful implementation of the prompts and discover likely responses to the proposed messages, in order to further inform prompt development and aid refinement of prompts. GP's were asked questions regarding their views, expectations, acceptability and feasibility of prompts. The semi-structured interview was conducted after showing GPs the initial paper-based versions of the prompts.

Think-aloud interviews were then conducted to study reactions to the prompts. GPs were asked to explore and try out the features of the prompts freely as they would if the messages had appeared during a consultation and say aloud what they were thinking and feeling about each feature. GPs were also prompted to reveal which functions were most/least useful and why.

### Materials

A series of prompts was designed to a) promote adherence to antibiotic prescribing recommendations in accordance with the NICE guidelines [32] (promote no antibiotic prescribing, or delayed antibiotic prescribing, instead of the immediate prescription of antibiotics where appropriate for RTI) and b) promote adherence to recommendations from the Intercollegiate Stroke Working Party for secondary prevention of stroke. The prompts were designed to remind GPs of the recommended behaviour, convince them it will be beneficial and assist them with implementation.

Prompts (for both RTI and stroke) were created drawing on aspects of Social Cognitive Theory [23]. The components of the theory which were targeted included, environment, outcome expectancies, and self-efficacy. Messages were designed to provide a controllable and supportive environment, promote positive outcome expectancies and increase self-efficacy.

The GP's environment was modified to provide support for guideline adherence, in that prompts were designed to appear on the GP's computer screen during a consultation for RTI or stroke (for the intervention trial which follows, prompts will automatically appear at appropriate consultations based on electronic condition read codes). This environment was designed to create maximum perceived controllability. The prompts appearing were controllable in terms of the range of functions and options available for GPs to select. The GP could therefore control if any information appeared, and the specific information which would be presented. All functions were supportive in terms of the messages and information to help the GP follow the guideline behaviour.

Outcome expectancies were addressed in the RTI prompts by presenting evidence that severity and duration of illness, as well as the risk of further complications, would not generally be increased by withholding an antibiotic prescription. Outcomes relating to concerns about patient expectations for antibiotics were addressed by presenting evidence suggesting that patients not prescribed antibiotics may be less likely to re-consult and believe antibiotics to be effective in future. Stroke prompts promoted positive outcome expectancies by emphasising the patient's reduced risk of suffering a further stroke if the GP followed the guidelines.

Techniques used to increase self-efficacy included elements of verbal persuasion and modelling. Verbal persuasion involved 'positive encouragement' in that GPs were told directly what they could do (e.g. "You can.."). For RTI, GPs were also given encouragement as to what actions they could take ('Instead of prescribing now you could...'). Verbal persuasion was only used to a minimal level in prompts due to lack of space and need for information to be concise as the GP would be viewing them during a consultation. The prompts also used 'modelling', by presenting evidence of the effect that performing the recommended behaviour has had on other patients. This use of 'modelling' in messages was rather implicit and brief, due to the nature of prompts in their presentation in a time limited environment.

The development process also involved close consultation with a working group of general practitioners and experts in the area of stroke prevention and RTI. The prompts were developed to form a series of electronic messages which would pop-up on the GP computer screen during a relevant consultation. Prompts were initially produced in a paper based form, with each sheet representing a screen. Prompts were refined and improved as interviews progressed based on feedback provided. Final prompt content included a reminder of the guideline, a summary of evidence relating to the guideline and the option to print a patient information sheet. After 22 interviews the prompts were developed into a prototype html-based format, which represented the way they would function in practice.

**Figure 1:**
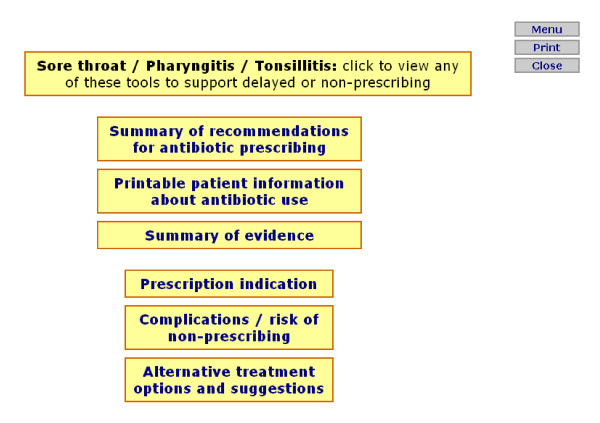
**Example of menu option screen for respiratory tract infection prompts (for sore throat/pharyngitis/tonsillitis)**.

**Figure 2:**
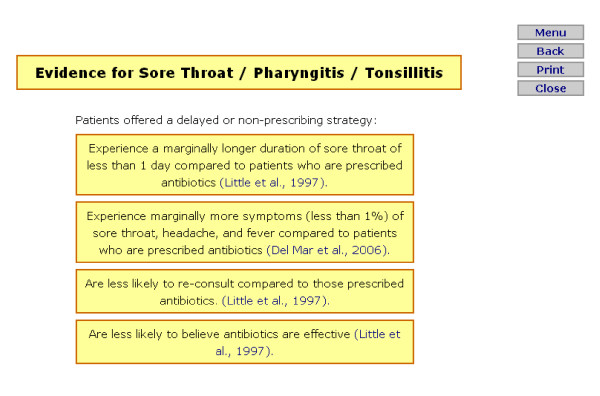
**Example of 'Summary of evidence' screen for respiratory tract infection prompts (for sore throat/pharyngitis/tonsillitis)**.

**Figure 3:**
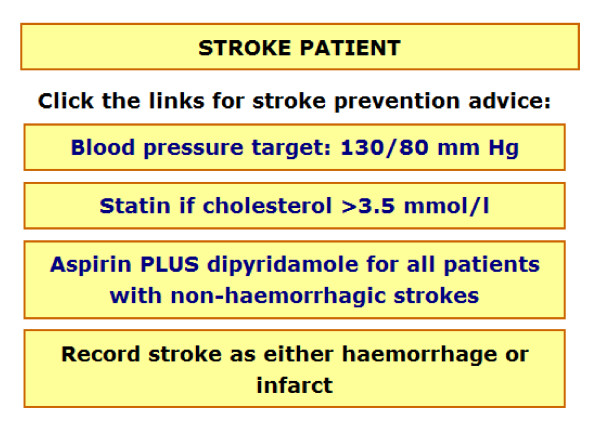
**Example of menu option screen for stroke prompts (showing all guidelines)**.

**Figure 4:**
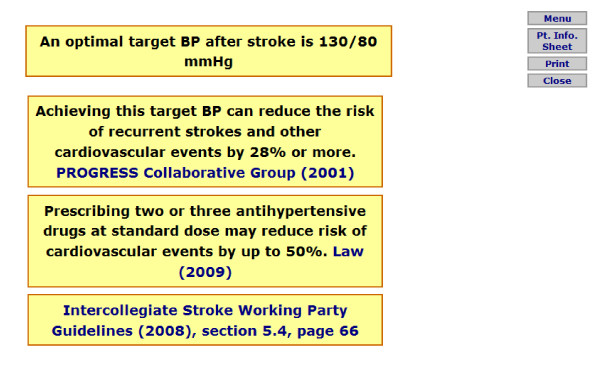
**Example of guideline screen for stroke prompts (for blood pressure target)**.

Prior to information appearing, RTI prompts first ask the GP to select which type of RTI they would like to view specific information for, these conditions are separated according to the NICE guidelines (sore throat/pharyngitis/tonsillitis, cough/bronchitis, otitis media, rhinosinusitis, and the common cold). A menu page then appears presenting all pages available to select and view (this is identical for each condition, however information appearing within each selection presents evidence specific to the condition). Figure 1 presents an example menu page, and figure 2 presents an example content page for the 'summary of evidence' option. For the stroke prompts, a menu page is first presented offering a selection of three guidelines (figure 3), each guideline page then provides information and further options relating to the guideline selected, an example of this in relation to the blood pressure guideline can be seen in figure 4.

### Analysis

Inductive thematic analysis [37] was conducted on all transcripts to determine likely responses to the prompts and identify factors involved in the decision to use the prompts and adhere to the guidelines. Analysis began after the first interview had been conducted and continued throughout data collection for all interviews conducted. Following immersion in the transcripts commonly occurring patterns and prominent themes were identified in the data and labelled with codes. Each code label referred to the operationalisation of the theme content. A coding manual was developed containing the label, a definition of each theme, positive examples from the interview transcripts, and possible exclusions. The coding manual was refined as more data became available, the continuing process involved themes being linked, grouped, moved, re-labelled, added and removed to produce a set of themes and coding manual which adequately fit and thoroughly explained the data. The coding was initially conducted by one author (L.M), themes and codes were then discussed with a second author (L.Y) and adjustments made where appropriate based on this discussion. Following this, inter-rater agreement was then reached on all codes.

## Results

Five themes emerged from the interviews, relating to the decision to use prompts and adhere to guidelines. Sub-themes were identified within each theme and are presented in Table 1. Themes were noted as being common across all interviews and did not differ across practice characteristics.

**Table 1:** Themes and sub-themes identified in GP interviews.

Themes	Sub-themes
Perceptions of role of prompts	-Rejection of enforcement and approval for choice
	-Acceptance of support tool
	
Patient outcomes	-Assistance in persuading patients
	-Perceived clinical appropriateness
	
Prescriber differences	-Willingness to use prompts
	-Useful for inexperienced staff
	
Accessibility and presentation of prompts	-Usability
	-Optimal information presentation
	-Tailored information
	-Provision of additional features
	
Acceptability of guidelines	-Caution about guideline differences
	-Credibility of source

### Development of prompts

The prompts were refined throughout the interview process based on continuing feedback. Early interviews provided many constructive criticisms and suggestions for change. Key changes and adaptations made to the prompts and the main themes which informed these can be seen in table 2. Once these features had been incorporated into the prompts, in the later interviews GPs expressed mainly positive comments about their use.

**Table 2:** Themes used to inform key changes in prompts.

Prompt Set	Key changes made	Relevant theme
ALL		
	Cancel option on all pages	Rejection of enforcement and approval for choice.
	Tabs to print, and return to menu added on every page	Usability.
	Menu page to select which information is viewed	Rejection of enforcement and approval for choice.
		Support tool
		
RTI		
	Alternative treatments tab merged into patient information sheet	Optimal information presentation.
		Usability.
	Advice on what to do removed from all additional information tabs	Rejection of enforcement and approval for choice.
		Optimal information presentation.
	Names of additional tabs made clearer	Usability.
		Optimal information presentation.
		
Stroke		
	Printable patient information sheet added	Assistance in persuading patients.
		Provision of additional features.
	Only guidelines relevant to individual patient appear	Tailored information.
	Link to Intercollegiate Stroke Working Party Guidelines	Credibility of source.
		Guideline conflict.

### Perceptions of role of prompts

The way in which the GP perceived the role of the prompts seemed to strongly affect whether they thought they would be likely to use them. The following sub-themes relate to the differing perceptions and how these were related to GPs' opinions of the prompts.

#### • Rejection of enforcement and approval for choice

The GPs reported strong rejection and opposition towards any technique perceived as being a method to enforce behaviour. However, there was a positive view and approval for methods perceived as allowing choice and control over prompt use.

"Whereas I've clicked into here voluntarily, I've not come into the room to be shouted at. It's just got to be, it's got to be in neutral. For me to take information in it's got to be my choice (....) But if I feel that it's actually behaviour modification... I won't, I won't probably go there." (P01)

#### • Acceptance of support tool

There was an acceptance and willingness to use the prompts if they were perceived as a support tool to aid the GP's own decision to follow guidelines.

"Well I think it's always you know, if you've decided on a delayed prescription, then the delayed prescription is now being supported by something useful" (P01)

"I think it will be ... a tool that is nice to know is there and more and more we have bits of paper in our drawers and they don't get pulled out cause we're too busy and something immediately accessible, that is linked to the patient's recent history, is quite useful." (P19)

"You could put this in the background somewhere. Something you could click on if you just want to remind yourself what the guidelines are." (P31)

### Anticipated patient outcomes

GPs' reported that their use of prompts would be influenced by expected patient outcomes. The sub-themes relate to patient expectations and patients medical need.

#### • Assistance in persuading patients

The prompts were seen as potentially providing assistance in persuading patients who may not be willing to adhere to advice recommended in the guideline.

"But if you want to try and persuade a patient who needs a bit of persuasion - you might like to try these screens." (P14)

#### • Perceived clinical appropriateness

Willingness to use the prompts was related to the perceived clinical need of the patient, and the specific benefit to the individual patient.

"Well I guess, you're always going to have some people who are not going to necessarily be - it's not really appropriate for them to be going from really ... aggressive medication just by having had a stroke, so if they've got incredible co-morbidity or just can't take more tablets or whatever, ...again it's a case of as long as you can ignore." (P08)

"But, of course, it wouldn't be appropriate, I guess, for a patient who's elderly, with chronic bronchitis and has an exacerbation. So that would be different." (P21)

### Prescriber differences

GPs reported that individual differences amongst practitioners was likely to influence the use of prompts. Sub-themes related to willingness to use prompts and differing staff influences.

#### • Willingness to use prompts

GPs predetermined willingness to use prompts determines whether or not prompts are used, regardless of content or potential benefit.

"I think it's partly going to depend on the GP's attitude towards it because if you've got somebody going, oh, this is ridiculous, it's on the screen, I don't use it, don't worry about it, then it's going to ... it won't be particularly helpful, but I think as long as you've got somebody who is into the idea and presents it properly, then, again, it's another useful way of objectifying it and saying, oh look, there's the evidence and I'm not just making it up." (P08)

#### • Useful for inexperienced staff

Inexperienced staff seen as likely to benefit from using the prompts.

"Yeah, I can see this being useful for registrars, new doctors, very useful for locums, actually, because we try to put together things for locums. We analyse our referral rates, for example, locums refer twice as many as we do." (P03)

### Accessibility and presentation of prompts

Participants suggested that usability issues concerning accessibility and presentation of information in prompts would influence the GP use of prompts. The sub-themes relate to various features of the prompts.

#### • Usability

All features of prompts should be easy to use and view, which will encourage their use.

"These would be very, very useful backups for us, just as long as there's not loads and loads of things that we have to wade through, and so, as long as it's quick and easy to understand, I think is ... very valuable, I think very good." (P06)

#### • Optimal information presentation

Evidence and information should be presented to include maximum detail in a minimal clear and concise format.

"I mean, there's a balance to be had, isn't there, between how much detail you offer and the accessibility of it and, clearly, it's a bit like doing slide presentations, you know, if you put too much stuff on, people think oh, I can't actually understand it and switch off." (P02)

#### • Tailored information

Information provided and prompts shown should be tailored as much as possible to the individual patient.

"I think it's great, I think it would be particularly good if it could be tailored to the person in front of me. Men, women, um you know, age, and co-morbidities maybe, but certainly age you know." (P01)

#### • Provision of additional features

Additional features should be added to the prompts to provide additional further benefit and support.

"The little bit of concern with me is that it's not quite integrated, I think this is getting better but we've got these other gaps, if you like, so the bit that's missing for me is the local information about referral routes, referral forms and so forth." (P07)

### Acceptability of guidelines

GP's attitudes to the prompts was related to the perceived acceptability of guidelines. Sub-themes relate to guideline differences and source.

#### • Caution about guideline differences

If GPs are aware of differences across guidelines, they are more likely to be cautious about using the prompts.

"Well, who do we follow, NICE or QOF [Quality and outcomes framework], that's the thing. You'll always get conflict. Some of us follow QOF because it's ... that's what we get paid for, so you've got a conflict really, but is it the best thing for the patient?" (P04)

#### • Credibility of source

Participants stated that they would be comfortable using prompts if the guideline was perceived as coming from a credible source.

"Yes. Seeing the Royal College of Physicians and the Stroke Working Party is enough, really. Yes. I'd look at that and think, oh, we should be doing that." (P19)

## Discussion

This study drew on social cognitive theory to develop prompts for two contrasting conditions one acute and self limiting and one involving secondary prevention. Analysis of data from interviews with GPs identified five key themes that GPs reported as likely to influence willingness to use prompts and adhere to guidelines. The themes were used to refine and adapt the original prompts, and led to the addition of features such as: printable patient information sheets; increased choice over information viewed; the option to cancel prompts; tailoring advice to patient characteristics; clearer information presentation, and other improvements to usability. Once these features had been incorporated GPs expressed generally positive views of the prompts.

The most important influence on GP attitudes appeared to be 'perception of the role of prompts'. GPs reported that if they felt that the guidelines or prompts were being enforced, they would develop a negative attitude towards them and be unlikely to use them constructively. However, if the GPs felt that they had control to choose to use the prompt and that it was supporting them, they would be likely to use it. This finding is consistent with the notion that control of environment plays an important role in successful behaviour change, which is an aspect of social cognitive theory [23] that had been included in the intervention development. In this instance, the GP was controlling the environment in terms of which prompts appeared when selected, and had ability to cancel the prompts if required. This finding is also consistent with self-determination theory [38], which argues that motivation towards a behaviour is strongest if an individual feels that they are acting autonomously, rather than responding to external influences. In relation to the intervention, GPs were autonomously choosing to view specific prompts and were not being forced to view set screens and messages.

A further factor which appeared to strongly influence the GPs' opinions of whether they were likely to use the prompts was anticipated patient outcomes. GPs reported that they would be more likely to use the prompts with patients who they felt needed persuasion to follow the guideline advice, and with patients who they felt it was clinically appropriate (e.g. unlikely to develop complications). This finding is consistent with the concept of outcome expectancies proposed in social cognitive theory [23] and used in the development of the intervention. In this case, GPs reported the need to reduce the negative outcomes of the patient being dissatisfied with the advice or experiencing further medical problems. These findings are also consistent with those of [27] who reported that both perceived medical need and perceived patient pressure had a significant effect in antibiotic prescribing decisions. Previous research has also consistently identified GP concerns over medical complications and negative medical consequences as major influences in prescribing decisions [35,39] which are prioritised over worries about antibiotic resistance in the case of RTIs [40].

Acceptability of the guidelines was also identified as influencing GPs' willingness to use prompts and follow the guidelines. GPs reported that they would be more likely to follow the advice if the guideline source was perceived as credible, and the recommendations did not conflict with any other guidelines. These findings are consistent with those of [28], who found that both credibility of source and content of guidelines were related to GPs' willingness to follow prescribing guidelines over a variety of conditions. Guideline differences has also been previously reported as a factor which may contribute to both confusion and lack of guideline uptake in GPs [41].

Individual prescriber differences were reported as influencing GPs' decisions to use the prompts. Many participants expressed the view that inexperienced staff (including trainees, nurses and registrars) were more likely to benefit from the prompts and that GPs would be likely to use these if they were training others. Although not possible in the current study, an additional application of the prompts as a training guide could be further developed, with the aim to increase adherence to guidelines in inexperienced or new staff.

A limitation of this study relates to the feasibility of incorporating all findings and feedback into the intervention. The GPs highly valued simple information presentation and usability, which were incorporated into the prompts, but many also suggested adding a range of additional features to prompts (e.g. local service information, medication information). Since a wide and varying range of features were requested it was difficult to identify further features which would benefit the majority of GPs, without creating an intervention which would be complex and difficult to use. GPs also expressed a desire for information tailored to the individual patient. This feature was included in development, in that if a patient has been recorded as already meeting any of the stroke targets recommended, the prompt relating to this guideline would not appear. However, the range of tailored information which many GPs requested could not be implemented fully due to the complexity involved in creating software that would make different recommendations based on a large number of patient characteristics. Finally, although the study revealed a number of interesting factors which GPs' report as potentially being influential in their decision to adhere to the intervention and guideline behaviours, the study did not trial the intervention or record the GPs' actual use of the prompts in practice. To establish the benefit of the intervention in adherence to guidelines a trial is necessary recording actual GP behaviour and patient outcomes, and further investigating GP views of using the prompts in daily practice.

## Conclusions

The qualitative process of working with GPs to develop a computer delivered intervention to follow guidelines, successfully resulted in the creation of prompts which GPs approved of. The study identified a number of factors which GPs reported would encourage them to use computer delivered prompts and adhere to guidelines.

A key characteristic of an acceptable computer-delivered intervention appears to be that it should be perceived as a useful tool supporting GP practice, rather than as didactic advice.

## Competing interests

The authors declare that they have no competing interests.

## Authors' contributions

LM conducted the study management (including design, interviews and analysis). LY assisted in intervention development and use of qualitative research. PL advised on content for RTI. MA advised on general practitioner usability issues. MG conceived the study and advised on all aspects. All authors read and approved the manuscript.

## Pre-publication history

The pre-publication history for this paper can be accessed here:

http://www.biomedcentral.com/1471-2296/11/90/prepub

## References

[B1] Hoomans T Severens JL Evers SM Ament AJ Value for money in changing clinical practice: should decisions about guidelines and implementation strategies be made sequentially or simultaneously? Med Decis Making 2009 29 207 216 10.1177/0272989X08327397 19237645

[B2] Grimshaw JM Eccles MP Walker AE Thomas RE Changing physicians' behavior: what works and thoughts on getting more things to work J Contin Educ Health Prof 2002 22 237 243 10.1002/chp.1340220408 12613059

[B3] Grimshaw JM Shirran L Thomas R Mowatt G Fraser C Bero L Changing provider behavior: an overview of systematic reviews of interventions Med Care 2001 39 II2 45 10.1097/00005650-200108002-00002 11583120

[B4] Simpson SA Butler CC Hood K Cohen D Dunstan F Evans MR Stemming the Tide of Antibiotic Resistance (STAR): a protocol for a trial of a complex intervention addressing the 'why' and 'how' of appropriate antibiotic prescribing in general practice BMC Fam Pract 2009 10 20 10.1186/1471-2296-10-20 19309493 PMC2666645

[B5] Hrisos S Eccles M Johnston M Francis J Kaner EF Steen N Developing the content of two behavioural interventions: using theory-based interventions to promote GP management of upper respiratory tract infection without prescribing antibiotics BMC Health Serv Res 2008 8 11 10.1186/1472-6963-8-11 18194527 PMC2267186

[B6] Rosser WW McDowell I Newell C Use of reminders for preventive procedures in family medicine CMAJ 1991 145 807 814 1913409 PMC1335900

[B7] Grimshaw JM Russell IT Effect of clinical guidelines on medical practice: a systematic review of rigorous evaluations Lancet 1993 342 1317 1322 10.1016/0140-6736(93)92244-N 7901634

[B8] Shiffman RN Liaw Y Brandt CA Corb GJ Computer-based guideline implementation systems: a systematic review of functionality and effectiveness J Am Med Inform Assoc 1999 6 104 114 10094063 10.1136/jamia.1999.0060104 PMC61349

[B9] Schriger DL Baraff LJ Rogers WH Cretin S Implementation of clinical guidelines using a computer charting system. Effect on the initial care of health care workers exposed to body fluids JAMA 1997 278 1585 1590 10.1001/jama.278.19.1585 9370504

[B10] Durieux P Nizard R Ravaud P Mounier N Lepage E A clinical decision support system for prevention of venous thromboembolism: effect on physician behavior JAMA 2000 283 2816 2821 10.1001/jama.283.21.2816 10838650

[B11] Christakis DA Zimmerman FJ Wright JA Garrison MM Rivara FP Davis RL A randomized controlled trial of point-of-care evidence to improve the antibiotic prescribing practices for otitis media in children Pediatrics 2001 107 E15 10.1542/peds.107.2.e15 11158489

[B12] Johnston ME Langton KB Haynes RB Mathieu A Effects of computer-based clinical decision support systems on clinician performance and patient outcome. A critical appraisal of research Ann Intern Med 1994 120 135 142 8256973 10.7326/0003-4819-120-2-199401150-00007

[B13] Grimshaw JM Thomas RE MacLennan G Fraser C Ramsay CR Vale L Effectiveness and efficiency of guideline dissemination and implementation strategies Health Technol Assess 2004 8 iii 72 14960256 10.3310/hta8060

[B14] Richens Y Rycroft-Malone J Morrell C Getting guidelines into practice: a literature review Nursing Standard 2004 18 33 40 10.7748/ns2004.08.18.50.33.c3677 15384305

[B15] Garg AX Adhikari NK McDonald H Rosas-Arellano MP Devereaux PJ Beyene J Effects of computerized clinical decision support systems on practitioner performance and patient outcomes: a systematic review JAMA 2005 293 1223 1238 10.1001/jama.293.10.1223 15755945

[B16] Davis RL Wright J Chalmers F Levenson L Brown JC Lozano P A cluster randomized clinical trial to improve prescribing patterns in ambulatory pediatrics PLoS Clin Trials 2007 2 e25 10.1371/journal.pctr.0020025 17525793 PMC1876598

[B17] Flottorp S Oxman AD Havelsrud K Treweek S Herrin J Cluster randomised controlled trial of tailored interventions to improve the management of urinary tract infections in women and sore throat BMJ 2002 325 367 10.1136/bmj.325.7360.367 12183309 PMC117890

[B18] Kooij FO Klok T Hollmann MW Kal JE Decision support increases guideline adherence for prescribing postoperative nausea and vomiting prophylaxis Anesth Analg 2008 106 893 8, table 10.1213/ane.0b013e31816194fb 18292437

[B19] Grol R Grimshaw J From best evidence to best practice: effective implementation of change in patients' care Lancet 2003 362 1225 1230 10.1016/S0140-6736(03)14546-1 14568747

[B20] Eccles MP Grimshaw JM Johnston M Steen N Pitts NB Thomas R Applying psychological theories to evidence-based clinical practice: Identifying factors predictive of managing upper respiratory tract infections without antibiotics Implement Sci 2007 2 26 10.1186/1748-5908-2-26 17683558 PMC2042498

[B21] Michie S Johnston M Abraham C Lawton R Parker D Walker A Making psychological theory useful for implementing evidence based practice: a consensus approach Qual Saf Health Care 2005 14 26 33 10.1136/qshc.2004.011155 15692000 PMC1743963

[B22] Michie S Fixsen D Grimshaw JM Eccles MP Specifying and reporting complex behaviour change interventions: the need for a scientific method Implement Sci 2009 4 40 10.1186/1748-5908-4-40 19607700 PMC2717906

[B23] Bandura A Self-efficacy: toward a unifying theory of behavioral change Psychol Rev 1977 84 191 215 10.1037/0033-295X.84.2.191 847061

[B24] Bandura A Social cognitive theory: an agentic perspective Annu Rev Psychol 2001 52 1 26 10.1146/annurev.psych.52.1.1 11148297

[B25] Bandura A Social cognitive theory of self-regulation Organizational Behavior and Human Decision Processes 1991 50 248 287 10.1016/0749-5978(91)90022-L

[B26] Glanz K Rimer BK Lewis FM Health Behavior and Health Education. Theory, Research and Practice 2002 San Fransisco: Wiley & Sons

[B27] Little P Dorward M Warner G Stephens K Senior J Moore M Importance of patient pressure and perceived pressure and perceived medical need for investigations, referral, and prescribing in primary care: nested observational study BMJ 2004 328 444 10.1136/bmj.38013.644086.7C 14966079 PMC344266

[B28] Rashidian A Eccles MP Russell I Falling on stony ground? A qualitative study of implementation of clinical guidelines' prescribing recommendations in primary care Health Policy 2008 85 148 161 10.1016/j.healthpol.2007.07.011 17767976

[B29] Campbell M Fitzpatrick R Haines A Kinmonth AL Sandercock P Spiegelhalter D Framework for design and evaluation of complex interventions to improve health BMJ 2000 321 694 696 10.1136/bmj.321.7262.694 10987780 PMC1118564

[B30] Francis N Wood F Simpson S Hood K Butler CC Developing an 'interactive' booklet on respiratory tract infections in children for use in primary care consultations Patient Educ Couns 2008 73 286 293 10.1016/j.pec.2008.07.020 18723306

[B31] Lewin S Glenton C Oxman AD Use of qualitative methods alongside randomised controlled trials of complex healthcare interventions: methodological study BMJ 2009 339 b3496 10.1136/bmj.b3496 19744976 PMC2741564

[B32] National Institute for Health and Clinical Excellence Prescribing of antibiotics for self limiting respiratory tract infections in adults and children in primary care 2008 National Institute for Health and Clinical Excellence. London 21698847

[B33] Rudd AG Lowe D Hoffman A Irwin P Pearson M Secondary prevention for stroke in the United Kingdom: results from the National Sentinel Audit of Stroke Age Ageing 2004 33 280 286 10.1093/ageing/afh107 15082434

[B34] Little P Williamson I Warner G Gould C Gantley M Kinmonth AL Open randomised trial of prescribing strategies in managing sore throat BMJ 1997 314 722 727 9116551 10.1136/bmj.314.7082.722 PMC2126131

[B35] Kumar S Little P Britten N Why do general practitioners prescribe antibiotics for sore throat? Grounded theory interview study BMJ 2003 326 138 10.1136/bmj.326.7381.138 12531847 PMC140007

[B36] Intercollegiate Stroke Working Party National Clinical Guidelines for Stroke 2008 Third London: Royal College of Physicians

[B37] Joffe H Yardley L Marks D, Yardley L Content and thematic analysis Research Methods for Clinical and Health Psychology 2004 London: Sage 56 68

[B38] Deci EL Ryan RM Berkowitz L The empirical exploration of intrinsic motivational processes Advances in experimental social psychology 1980 New York: Academic press 39 80 10.1016/S0065-2601(08)60130-6

[B39] Wood F Simpson S Butler CC Socially responsible antibiotic choices in primary care: a qualitative study of GPs' decisions to prescribe broad-spectrum and fluroquinolone antibiotics Fam Pract 2007 24 427 434 10.1093/fampra/cmm040 17728289

[B40] Simpson SA Wood F Butler CC General practitioners' perceptions of antimicrobial resistance: a qualitative study J Antimicrob Chemother 2007 59 292 296 10.1093/jac/dkl467 17110392

[B41] Calderon C Rotaeche R Etxebarria A Marzo M Rico R Barandiaran M Gaining insight into the Clinical Practice Guideline development processes: qualitative study in a workshop to implement the GRADE proposal in Spain BMC Health Serv Res 2006 6 138 10.1186/1472-6963-6-138 17059600 PMC1626459

